# Postoperative remodeling and developmental improvement of femoral trochlear dysplasia in pediatric habitual patellar dislocation

**DOI:** 10.3389/fped.2026.1869246

**Published:** 2026-07-09

**Authors:** Yanpeng Xu, Shaoqi Yang, Haiyang Jiang, Lin Huang, Shiqi Wan, Jiayao Song, Zhenqun Zhao, Chao Feng

**Affiliations:** 1 Department of Pediatric Surgery, Affiliated Hospital of Zunyi Medical University, Zunyi, China; 2 Department of Pediatric Surgery, Guizhou Children’s Hospital, Zunyi, China; 3 Department of Pediatric Sports Medicine, Beijing Jishuitan Hospital, Capital Medical University, Beijing, China

**Keywords:** children, habitual patellar dislocation, trochlear development, trochlear dysplasia, trochlear morphology

## Abstract

**Background:**

Trochlear dysplasia (TD) is a major contributor to habitual patellar dislocation (HPD). However, studies on femoral trochlear remodeling after HPD surgery in children under 10 years old are limited and have small sample sizes.

**Purpose:**

This study evaluated postoperative improvements in trochlear morphology and identified factors associated with trochlear development in surgically treated children <10 years old with HPD.

**Methods:**

Clinical data of patients younger than 10 years with HPD treated between 2017 and 2023 were retrospectively reviewed. MRI was used to measure femoral sulcus angle (SA), relative trochlear depth (RTD), lateral trochlear inclination (LTI), and trochlear facet angle (TFA). Trochlear development was assessed via Dejour classification. Paired t-test and Fisher's exact test compared pre- and postoperative differences. Patients were divided into normal and poor prognosis groups; correlations between preoperative indicators, follow-up time, and postoperative TD were analyzed. GLMM logistic regression identified influencing factors, and ROC curve determined optimal cut-off values of relevant factors.

**Results:**

A total of 19 cases (24 knees) were included, with a mean age of 79.21 ± 31.26 months and mean follow-up of 35.92 ± 15.24 months. Postoperatively, SA significantly decreased (*t* = 8.756, *P* < 0.001), while RTD, LTI, and TFA significantly increased (*Z* = −3.857, *t* = −6.957, *t* = −7.790; all *P* < 0.001). Preoperatively, 2 knees had normal trochlear development and 22 had TD (Dejour type A:13, B:8, C:1); postoperatively, 10 knees normalized and 14 had persistent TD (type A:12, B:2), showing significant improvement (*P* = 0.008). Preoperative Age differed significantly between prognosis groups (*F* = 7.341, *P* = 0.015). Preoperative Age and SA were positively correlated with postoperative TD (*r* = 0.497, *P* = 0.014; *r* = 0.421, *P* = 0.040), while preoperative RTD and LTI were negatively correlated (*r* = −0.415, *P* = 0.044; *r* = −0.427, *P* = 0.037). Preoperative Age was an independent risk factor for postoperative TD (*OR* = 1.105, *P* = 0.004), with an optimal cut-off of 73.5 months (better prognosis in children <73.5 months).

**Conclusion:**

Trochlear morphology significantly improves postoperatively in children <10 years old with HPD. Smaller preoperative Age and SA, and larger RTD and LTI, correlate with better postoperative outcomes; preoperative Age is an independent risk factor for poor trochlear development. Early surgery (age <73.5 months) is a favorable factor for obtaining a good prognosis in trochlear remodeling.

## Introduction

HPD is a common knee disorder in the pediatric population. Trochlear dysplasia (TD), typically characterized by a shallow, flattened, or convex trochlear groove, is one of the primary causative factors of HPD. Previous studies have documented trochlear morphological changes and trochlear remodeling after surgery in children with congenital or recurrent patellar dislocation (1, 2). However, evidence regarding postoperative trochlear remodeling in pediatric HPD remains limited, with existing studies featuring small sample sizes and predominantly relying on computed tomography (CT) for morphological assessment (1, 3, 4). In young children, especially those under 6 years old, open physes and thick trochlear articular cartilage may lead to substantial errors when using CT to evaluate bony anatomy alone. Magnetic resonance imaging (MRI) enables more accurate assessment of cartilaginous trochlear morphology and possesses unique advantages in evaluating trochlear remodeling (5–7). Therefore, this retrospective study included 19 pediatric patients (24 knees) under 10 years of age with HPD. Using MRI measurements, we aimed to investigate postoperative changes in trochlear morphology and identify factors influencing trochlear development, thereby providing a theoretical basis for predicting postoperative trochlear growth and remodeling.

## Methods

### Study population

Clinical data of pediatric patients diagnosed with HPD were retrospectively collected from Beijing Jishuitan Hospital, Capital Medical University between January 2017 and December 2023. Demographic and clinical characteristics, including age, gender, affected side, surgical procedure, and follow-up duration, were extracted and systematically analyzed. The inclusion criteria were as follows: 1) Age ranging from 0 to 10 years at the time of surgery. 2) Complete preoperative and postoperative MRI data available for review. 3) A minimum follow-up period of 24 months after the index operation. 4) Written informed consent obtained from the legal guardians of all enrolled children. The exclusion criteria were as follows: 1) Diagnosis of other types of patellar dislocation, including acute traumatic patellar dislocation, congenital patellar dislocation, and recurrent patellar dislocation; 2) A history of prior knee surgery before the index operation for HPD; 3) Presence of congenital bone or soft tissue disorders, concurrent infection, or neoplastic lesions involving the knee joint.

This study was conducted in strict accordance with the principles of the Declaration of Helsinki and was approved by the Ethics Committee of Beijing Jishuitan Hospital, Capital Medical University (Approval No.: 202201-26).

### Surgical techniques

Lateral retinacular release was performed in cases where a contracted lateral retinaculum precludvented adequate patellar reduction (lateral glide test < Ⅱ°). Lateral half-patellar tendon transposition was indicated when the tibial tubercle – trochlear groove (TT-TG) distance — defined as the horizontal distance between the anterior apex of the tibial tubercle and the deepest point of the femoral trochlear cartilage — exceeded 20 mm, or when the Q angle was ≥ 20°. Medial patellofemoral ligament (MPFL) reconstruction using adductor magnus tendon transfer was performed in all patients aged 6 years and older.

### Measurement methods

To evaluate morphological improvements of the trochlea after surgery, magnetic resonance imaging (MRI) was used to measure preoperative and postoperative parameters including the SA, RTD, LTI, and TFA. Concurrently,trochlear dysplasia was simultaneously assessed according to the Dejour classification system (8, 9). Detailed measurement protocols are described in the following section.

**Figure 1:**
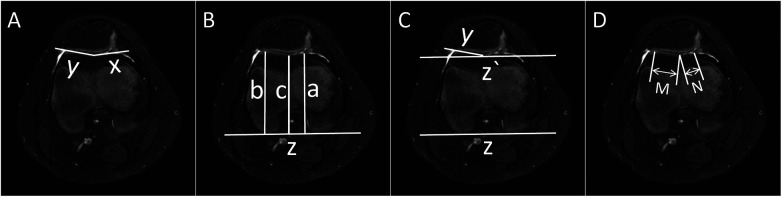
MRI measurements of femoral trochlear morphological parameters. **(A)** SA was defined as the angle between Line x and y, where Line x passed through the highest point of the medial condyle and the lowest point of the trochlear sulcus, and Line y passed through the highest point of the lateral condyle and the lowest point of the trochlear sulcus. **(B)** RTD was defined as the ratio of trochlear sulcus depth to the maximum anteroposterior diameter of the lateral femoral condyle, which was calculated by the formula [(a + b)/2−c]/b. **(C)** LTI was defined as the angle between Line y and Line z′; Line z′ was parallel to the posterior condylar tangent (Line z) and passes through the lowest point of the trochlear sulcus. **(D)** TFA was defined as the length ratio of the medial trochlear facet to the lateral trochlear facet, which was calculated as N/M.

All measurements were performed on the axial MRI slice at the maximal anteroposterior diameter of the lateral femoral condyle (10), as illustrated in Figure 1. The SA was defined as the angle formed between the medial and lateral trochlear articular facets. Specifically, Lines x and y were drawn through the highest points of the medial and lateral condyles, and the lowest point of the trochlear sulcus, respectively; the angle between lines x and Line y was recorded as SA (Figure 1A). The RTD was calculated as the ratio of trochlear sulcus depth to the maximal anteroposterior diameter of the lateral femoral condyle. A tangent line (Line z) was drawn along the posterior margin of the femoral condyles. The heights of the medial condyle (a), lateral condyle (b), and trochlear sulcus (c, defined as the shortest distance from the lowest point of the trochlear sulcus to Line z) were measured. The formula for RTD was defined as [(a + b)/2−c]/b (Figure 1B). The LTI was defined as the angle between the line along the lateral trochlear facet and the posterior condylar line. Line z′ was drawn parallel to Line z through the lowest point of the trochlear sulcus; the angle between line y and line z′ was defined as LTI (Figure 1C). The TFA was represented as the length ratio of the medial trochlear facet to the lateral trochlear facet. Segments N and M were drawn from the highest points of the medial and lateral condyles to the lowest point of the trochlear sulcus, respectively. The TFA was calculated as the length ratio N/M (Figure 1D).

### Statistical analysis

Statistical analyses were performed using SPSS 29.0 software. All observers received standardized pre-measurement training. Pilot interobserver testing revealed minimal measurement differences, so only Intraclass correlation coefficient (ICC) was used to evaluate the intraobserver test-retest reliability. For comparisons of preoperative and postoperative measurements, normally distributed quantitative data were expressed as mean ± standard deviation (x¯ ± s) and analyzed with the paired t-test. Non-normally distributed data were presented as median (interquartile range) [M (Q1, Q3)] and assessed using the Wilcoxon signed-rank test. Categorical variables were compared using Fisher's exact test. Linear mixed-effects models (LMM) and generalized estimating equations (GEE) were applied to normally and non-normally distributed data, respectively, to compare variables between the two prognostic and age groups. Correlation analyses were performed using the Pearson correlation coefficient for normally distributed data and the Spearman rank correlation coefficient for non-normally distributed data. Generalized linear mixed-effects model logistic regression (GLMM logistic regression) was used to identify independent risk factors for postoperative trochlear dysplasia. Receiver operating characteristic (ROC) curves were constructed to determine the optimal cutoff values of parameters associated with trochlear remodeling. In addition, the postoperative improvement degrees of each parameter were calculated as follows: the improvement rates of SA, LTI and TFA were defined as∣SA_postop_−SA_preop_∣/ SA_preop_, ∣LTI_postop_−LTI_preop_∣/ LTI_preop_,∣TFA_postope_−TFA_preop_∣/ TFA_preop_, respectively. The postoperative improvement degree of RTD was calculated as RTD_postop_−RTD_preop_. All analyses were performed with a 95% confidence interval (CI), and a *P* < 0.05 was considered statistically significant.

## Results

**Figure 2:**
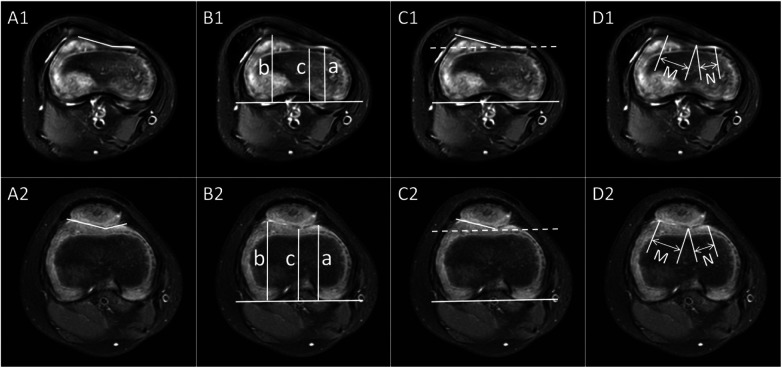
Changes in trochlear morphology following surgical treatment for right HPD in a 6-year-old male patient. **(A1–D1)** Represent preoperative MRI measurements of the SA, RTD, LTI and TFA, respectively. **(A2–D2)** Represent MRI measurements of the SA, RTD, LTI and TFA at 26 months postoperatively, respectively.

A total of 19 patients (24 knees) were enrolled in this study, consisting of 9 males (11 knees) and 10 females (13 knees). Among these patients, 9 cases involved the left knee, 4 cases involved the right knee, and 5 cases had bilateral knee involvement. The mean age was 79.21 ± 31.26 months (range, 18–120 months), with a mean follow-up duration of 35.92 ± 15.24 months (range, 24–69 months). At the final follow-up, all patients achieved a normal range of motion of the knee joint, and no recurrence of patellar dislocation was observed in any patient. For the intraobserver test-retest reliability of MRI-derived measurements, all parameters yielded ICC values above 0.9, indicating excellent repeatability (Table 1). Compared with the preoperative SA (165.893 ± 9.714°), RTD [0.037 (0.026, 0.057)], LTI (12.294 ± 4.635°), and TFA (0.567 ± 0.104), the postoperative SA was significantly decreased to 151.759 ± 9.771°, with a statistically significant difference (t = 8.756, *P* < 0.001). Postoperatively, RTD [0.072 (0.055, 0.096)], LTI (18.587 ± 5.708°), and TFA (0.701 ± 0.102) were significantly increased, and the differences were statistically significant (*Z* = −3.857, *t* = −6.957, *t* = −7.790; all *P* < 0.001) (Table 2, Figure 2). In addition, trochlear dysplasia was re-evaluated using the Dejour classification system. Preoperatively, 2 knees exhibited normal trochlear morphology, while 22 knees had trochlear dysplasia, including 13 knees of Dejour A, 8 knees of Dejour B, and 1 knee of Dejour C. Postoperatively, 10 knees achieved normal trochlear development, and 14 knees still had trochlear dysplasia, consisting of 12 knees of Dejour A and 2 knees of Dejour B. The results indicated that postoperative trochlear morphology was significantly improved, with a statistically significant difference (*P* = 0.008) (Table 3).

**Table 1:** Intraobserver test-retest reliability of MRI measurements.

Measurements	ICC	95% CI	*F*	*P*
SA/Preop	0.974	0.951–0.988	115.206	<0.001
RTD/Preop	0.981	0.962–0.991	152.574	<0.001
LTI/Preop	0.932	0.871–0.967	40.827	<0.001
TFA/Preop	0.965	0.933–0.984	80.892	<0.001
SA/Postop	0.961	0.924–0.981	71.159	<0.001
RTD/Postop	0.923	0.857–0.963	36.339	<0.001
LTI/Postop	0.984	0.901–0.975	53.626	<0.001
TFA/Postop	0.962	0.927–0.982	73.754	<0.001

**Table 2:** Comparison of MRI-measured indicators between the preoperative and postoperative periods.

Parameter	Preop	Postop	*t*/*Z*	*P*
SA(°)	165.893 ± 9.714	151.759 ± 9.771	8.756	<0.001
RTD	0.037 (0.026, 0.057)	0.072 (0.055, 0.096)	−3.857	<0.001
LTI(°)	12.294 ± 4.635	18.587 ± 5.708	−6.957	<0.001
TFA	0.567 ± 0.104	0.701 ± 0.102	−7.790	<0.001

**Table 3:** Comparison of trochlear development preoperatively and postoperatively.

Time point	Trochlear development		*P*
	Normal	Dysplasia	
Preop	2	22	
Postop	10	14	0.008

**Figure 3:**
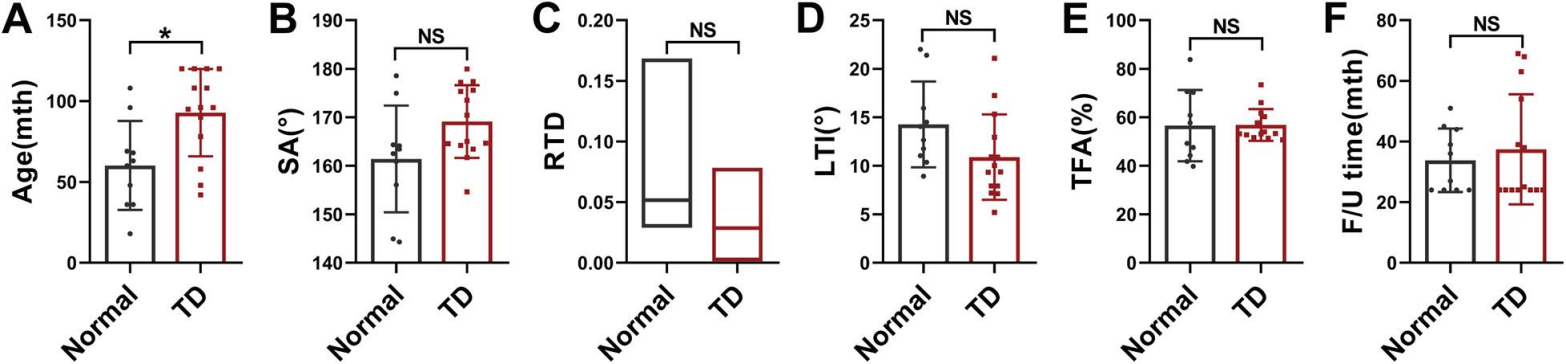
Comparison of preoperative indicators and postoperative F/U time between the normal group and the TD group. **(A)** Age, **(B)** SA, **(C)** RTD, **(D)** LTI, **(E)** TFA, **(F)** F/U time. *P < 0.05.

**Figure 4:**
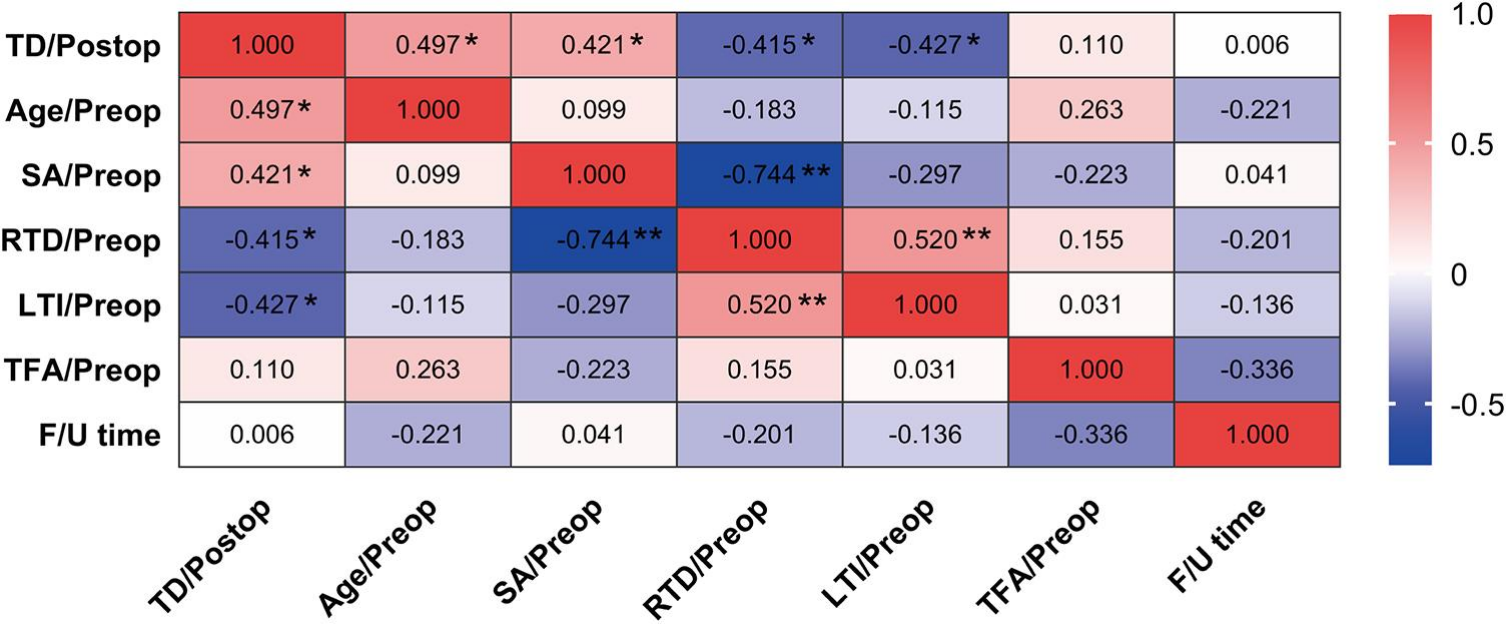
Correlation analysis of factors influencing postoperative trochlear dysplasia. * *P* < 0.05, ** *P* < 0.01.

Second, patients were stratified into two groups according to the prognostic outcomes of postoperative trochlear morphological changes: the normal trochlear development group (Normal group) and the trochlear dysplasia group (TD group). Differences in preoperative indicators, including Age, SA, RTD, LTI, TFA, as well as postoperative follow-up time (F/U time), were compared between the two prognostic groups. The results revealed a significant difference in preoperative age between the two prognostic groups (*F* = 7.341, *P* = 0.015). The mean preoperative age was 60.200 ± 27.51 months in the normal development group and 92.786 ± 26.993 months in the dysplasia group (Figure 3). Further analysis was conducted to explore the correlation between preoperative indicators (Age, SA, RTD, LTI, TFA), postoperative F/U time, and poor prognosis. The results revealed that preoperative Age and SA were positively correlated with postoperative trochlear dysplasia (Age: *r* = 0.497, *P* = 0.014; SA: *r* = 0.421, *P* = 0.040), whereas preoperative RTD and LTI were negatively correlated with postoperative trochlear dysplasia (RTD: *r* = −0.415, *P* = 0.044; LTI: *r* = −0.427, *P* = 0.037) (Figure 4). In addition, GLMM logistic regression analysis indicated that preoperative age was an independent risk factor for postoperative trochlear dysplasia (*P* = 0.004) (Table 4).

**Table 4:** GLMM logistic regression for predictors of postoperative trochlear dysplasia.

Variable	*B*	*SE*	*OR*	*95% CI*	*P*
Age	0.100	0.030	1.105	1.036∼1.178	0.004
SA	0.166	0.146	1.180	0.867∼1.605	0.272
RTD	19.422	46.391	–	–	0.648
LTI	−0.207	0.245	0.813	0.485∼1.365	0.411
TFA	−4.017	8.744	–	–	0.652
F/U time	0.065	0.064	1.068	0.932∼1.223	0.324

Complete data separation for RTD and TFA resulted in unstable estimates and extremely wide confidence intervals.

**Figure 5:**
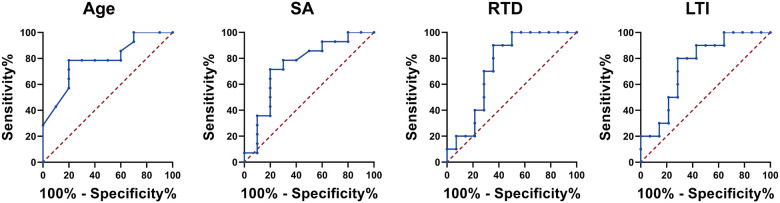
ROC curves of each indicator.

**Figure 6:**
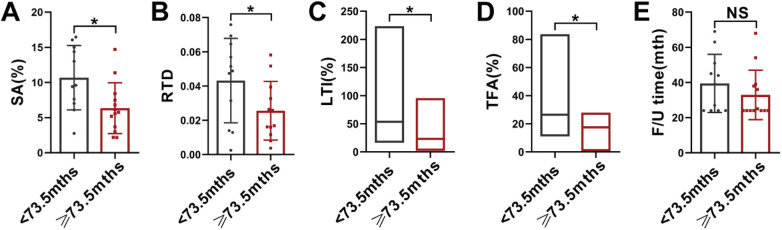
Comparison of postoperative improvement degrees of each indicator between two groups of children stratified by preoperative age. **(A)** SA, **(B)** RTD, **(C)** LTI, **(D)** TFA, **(E)** F/U time. *P < 0.05.

Furthermore, to further elucidate the impact of preoperative Age on poor prognosis, the cut-off values of each indicator were analyzed. The results showed that the area under the curve (AUC) of preoperative Age was greater than 0.700, which confirmed the reliability of the analysis findings, and the optimal cut-off value of Age was 73.5 months. Additionally, the AUC values of SA, RTD, and LTI were also greater than 0.700, with their optimal cut-off values being 164.442°, 0.034, and 10.973°, respectively (Figure 5, Table 5). To verify the optimal cut-off value of age (73.5 months), the children were divided into two groups based on their preoperative age: the <73.5 months group and the ≥73.5 months group. Differences in the improvement degrees of postoperative SA, RTD, LTI, TFA, and follow-up (F/U) time between the two groups were compared. The results revealed that the improvement degrees of SA, RTD, LTI, and TFA in the <73.5 months group were [10.685 ± 4.574%, 4.319 ± 2.466, 53.675 (37.259, 94.707)%, 26.482 (12.812, 55.450)%], respectively, whereas those in the ≥73.5 months group were [6.349 ± 3.610%, 2.367 ± 1.775, 23.139 (16.077, 59.513)%, 17.471 (9.611, 25.628)%], respectively. The improvement degrees in the <73.5 months group were significantly superior to those in the ≥73.5 months group, with statistically significant differences (SA: *F* = 6.743, *P* = 0.016; RTD: *F* = 4.993, *P* = 0.036; LTI: *Wald χ^2^* = 5.704, *P* = 0.007; TFA: *Wald χ^2^* = 12.972, *P* < 0.001). In contrast, no significant difference was observed in F/U time between the two groups (*F* = 1.099, *P* = 0.306) (Figure 6). We further compared the differences in prognostic outcomes between the two groups of children. The results demonstrated that the prognostic outcomes of children in the <73.5 months group were significantly better than those in the ≥73.5 months group, with a statistically significant difference (*P* = 0.011) (Table 6).

**Table 5:** ROC curve analysis for determining the optimal cut-off values of each indicator.

Variable	AUC	*95% CI*	*P*	Sensitivity	Specificity	Cut-off value
Age(mth)	0.789	0.605∼0.974	0.018	0.786	0.800	73.500
SA(°)	0.746	0.533∼0.960	0.043	0.714	0.800	164.442
RTD	0.743	0.541∼0.945	0.046	0.900	0.643	0.034
LTI(°)	0.750	0.552∼0.948	0.040	0.800	0.714	10.973
TFA	0.564	0.282∼0.847	0.598	1.000	0.500	0.499
F/U time(mth)	0.504	0.266∼0.741	0.977	0.286	1.000	52.500

**Table 6:** Comparison of postoperative prognosis between two age-stratified pediatric groups.

Trochlear morphology	Trochlear development		*P*
	<73.5mths	73.5mths	
Normal	8	3	
Dysplasia	2	11	0.011

## Discussion

Pediatric HPD is a relatively common developmental disorder characterized by patellofemoral joint instability and medial soft tissue insufficiency (11, 12). Among the various etiological factors underlying this condition, trochlear dysplasia is widely acknowledged as one of the most crucial pathogenic determinants (7, 13–15). In patients with trochlear dysplasia, the trochlear groove exhibits abnormal flatness or even convexity, leading to the loss of effective osseous constraint on the patella. This structural abnormality impairs the patella's stability within the trochlear groove, making it susceptible to lateral displacement and subsequent dislocation. Consistent with this pathogenic mechanism, our study demonstrated that 17 out of 19 pediatric patients with HPD (89.47%) had varying degrees of trochlear dysplasia before surgical intervention. This result further confirms the pivotal role of trochlear development in the pathogenesis of pediatric patellar dislocation.

At present, the primary clinical examinations for assessing trochlear development include x-ray films, CT, and MRI. Additionally, there are reports regarding the application of ultrasound for examining infants aged ≤ 6 months (16). In the present study, we referred to the method described by Hou L (17) and conducted measurements on the MRI slice corresponding to the maximum anteroposterior diameter of the lateral femoral condyle. Notably, MRI can more accurately and authentically reflect the depth of the cartilaginous trochlear groove and the height of the lateral trochlear wall. Simultaneously, performing measurements on the slice with the maximum anteroposterior diameter of the lateral femoral condyle ensures the comparability of pre- and postoperative measurement data, thereby enhancing the reliability and validity of the study results. Among the measured indicators, we mainly evaluated the SA, RTD, LTI, and TFA, while trochlear groove depth (TGD) was not included. This exclusion was primarily due to significant variations in trochlear size development among children aged 0 to 10 years; using a fixed cutoff of TGD < 3 mm as an indicator of trochlear dysplasia fails to accurately reflect the trochlear developmental status in children of different age groups. Therefore, we adopted RTD, defined as the ratio of trochlear groove depth to the maximum anteroposterior diameter of the lateral femoral condyle. This indicator maintains uniformity when assessing trochlear groove depth development across children of different ages, ensuring the accuracy and comparability of our measurements. Thus, the MRI-measured indicators employed in this study can more authentically reflect the morphological development of the trochlea in pediatric HPD.

Through a comprehensive literature review, we found that: Hao K (1) conducted a study involving 48 children with patellar dislocation, without differentiating between the specific subtypes of patellar dislocation. Separately, Guo H (5) and Hou L (17) carried out research on 20 children with recurrent patellar dislocation (RPD) and 58 children (80 knees) with congenital patellar dislocation (CPD), respectively. Notably, only Benoit B (18) have reported improvements in postoperative trochlear development among 8 children (12 knees) with HPD aged 7–14 years. Collectively, these findings indicate that there is a paucity of studies investigating postoperative trochlear remodeling in children with HPD, and the study subjects in existing literature are mostly older, which precludes a comprehensive evaluation of overall trochlear remodeling following early surgical intervention. Thus, additional research is warranted to supplement and deepen the understanding of this field. Therefore, we enrolled 19 children (24 knees) with HPD under 10 years of age to further analyze postoperative trochlear morphological development and its associated influencing factors, which to a certain extent addresses the research gaps in this area.

Surgical intervention is the primary therapeutic approach for pediatric HPD (19, 20). Previous studies have demonstrated that for different subtypes of patellar dislocation, surgical intervention before epiphyseal closure can lead to a certain degree of improvement in postoperative trochlear development (1, 5, 18). However, a study by Monllau JC et al. revealed that short-term postoperative outcomes of patellar dislocation in older children had no significant impact on the patellofemoral joint, including the trochlear structure (21). To further confirm the improvement of trochlear morphology following surgical treatment for HPD, we performed surgical intervention on 19 children (24 knees) diagnosed with HPD. After a mean follow-up period of 35.92 ± 15.24 months, we found that the trochlear morphology of these patients was significantly improved. This finding indicates that the trochlear morphology of children with HPD exhibits good plasticity following surgical management, providing important clinical evidence for the efficacy of early surgical intervention in promoting trochlear remodeling. Although trochlear morphology was improved after surgical treatment for patellar dislocation, the present study found that not all cases achieved normal trochlear developmental levels. Specifically, 11 children (14 knees) still presented with trochlear dysplasia postoperatively, including 12 knees (50.00%) of Dejour A and 2 knees (8.33%) of Dejour B. To explore the underlying factors contributing to this phenomenon, we analyzed the correlation between preoperative indicators such as Age, SA, RTD, LTI, TFA, and F/U time and postoperative trochlear dysplasia. The results demonstrated that preoperative Age, SA, RTD, and LTI were closely correlated with postoperative trochlear development, and preoperative Age was identified as an independent risk factor affecting the improvement of postoperative trochlear morphology in children with HPD. This indicates that the age of children plays an important role in determining the outcome of postoperative trochlear development.

Currently, most studies investigating trochlear remodeling following surgical treatment for pediatric patellar dislocation have focused on the 7–14 years old age group (1, 5, 18). Hou L conducted a study on postoperative trochlear remodeling in children under 6 years old with CPD (17). However, our study suggests that children with HPD require early surgical intervention to achieve optimal improvement in trochlear development. Nevertheless, there are no relevant reports regarding the specific age threshold below which surgery can yield the best postoperative trochlear remodeling effect. Given that the trochlea has relatively limited growth potential in children over 10 years old (22), the present study enrolled children with HPD under 10 years old, aiming to determine the optimal cutoff age for surgical intervention to maximize postoperative trochlear remodeling. Therefore, we performed receiver operating characteristic ROC curve analysis between each of the preoperative indicators (Age, SA, RTD, LTI, TFA), postoperative F/U time and different prognostic outcomes. The results showed that the AUC of preoperative Age was the largest, indicating the highest credibility. Further analysis revealed that the optimal cut-off value of preoperative Age was 73.5 months (6 years and 1 month), which was verified. This indicates that children with HPD whose preoperative age is less than 73.5 months have a relatively better trochlear prognosis outcome. This finding supplements the clinical research on the optimal age threshold for treating children with HPD and provides a certain basis for our suggestion that children with HPD should undergo surgical intervention before school age.

The limitations of this study are as follows: First, the sample size was small and follow-up period was short. No non-operative control group was included. Enrolment limited to patients with complete MRI follow-up may induce selection bias and weaken generalizability. We will address these shortcomings in future research. Second, all measurements used two-dimensional (2D) MRI. Three-dimensional (3D) MRI will be adopted to assess femoral trochlear morphology for more reliable morphological analysis (6, 23). Additionally, different surgical approaches across age groups may cause divergent patellofemoral joint biomechanics, an issue needing further exploration. Moreover, the adult-oriented Dejour classification has limitations in paediatric populations. Its subjective grading may reduce diagnostic reliability by neglecting paediatric trochlear development, and age-specific grading criteria should be established in future work.

In summary, the present study allows us to draw the following conclusions: 1) After surgical correction of patellar dislocation in children with HPD aged under 10 years, the developmental morphology of the trochlea can be significantly improved. 2) A smaller preoperative age and SA, as well as a larger RTD and LTI, are associated with better improvement in postoperative trochlear morphology. Among these indicators, the preoperative age is more closely related to the improvement degree of postoperative trochlear development and can serve as an independent key factor influencing trochlear development following HPD surgery. 3) This study suggests that for children with HPD under 10 years old, surgery performed before 73.5 months (approximately 6 years and 1 month) may be associated with better postoperative trochlear development.

## Data Availability

The raw data supporting the conclusions of this article will be made available by the authors, without undue reservation.
